# Respiratory Disorders Related to e-Waste Exposure among Workers in the Informal Sector in a Sub-Saharan African City: An Exposed Nonexposed Study

**DOI:** 10.1155/2022/9968897

**Published:** 2022-02-24

**Authors:** Ablo Prudence Wachinou, Nonvignon Marius Kêdoté, Geraud Padonou, Serge Adè, Joaquin Darboux, Mirlène Tohi, Arnauld Fiogbé, Julius Fobil, Gildas Agodokpessi

**Affiliations:** ^1^Faculty of Health Sciences, University of Abomey-Calavi, Cotonou, Benin; ^2^Centre National Hospitalier Universitaire de Pneumo-Phtisiologie, Cotonou, Benin; ^3^Department of Health Environment, Regional Institute of Public Health Comlan Alfred Quenum (IRSP-CAQ), University of Abomey-Calavi, Benin; ^4^Ecohealth Chair/Community of Practice Ecohealth for West and Central Africa (COPES-AOC), University of Abomey-Calavi, Benin; ^5^Faculty of Medicine, University of Parakou, Parakou, Benin; ^6^Department of Biological, Environmental, and Occupational Health Sciences, School of Public Health, West Africa GEOHealth Network, University of Ghana, Ghana

## Abstract

**Introduction:**

Exposure to electrical and electronic equipment waste (e-waste) has become a growing health concern. The objective of this study was to measure the effect of exposure to e-waste on respiratory symptoms and on lung function parameters in workers involved in informal recycling activities in Cotonou city, Benin.

**Methods:**

This was a cross-sectional study, in which exposed e-waste workers in Cotonou city were randomly selected. A matching nonexposed group based on age and sex was recruited from the general population. Respiratory symptoms were investigated using a questionnaire adapted from the British Medical Research Council's standardized respiratory questionnaire. Participants underwent lung function test using a portable spirometer (MIR SPIROBANK). Data were analyzed with STATA version 15 software.

**Results:**

The overall prevalence of respiratory symptoms in e-waste workers was statistically higher in the exposed group (33.1% vs. 21.6%; *p* = 0.027). Chest tightness (11.8% vs. 2.1%; *p* = 0.003) and breathlessness (6.8% vs. 1.4%; *p* = 0.018) were the most reported symptoms by e-waste workers. Lung function testing showed a higher proportion of disorders among e-waste workers (25.0% vs. 14.9%, *p* = 0.029), with a higher proportion of probable restrictive (10.8% vs. 2.7, *p* = 0.005) and mixed (4.1% vs. 0%, *p* = 0.013) ventilatory disorders. Handling or working with e-waste was found associated with a significant reduction in forced expiratory volume in one second (FEV1) by 0.4 L (95% CI: 0.3-0.6) and forced vital capacity (FVC) by 0.75 L (95% CI: 0.6-0.9) after adjustment for age, BMI, smoking habits, asthma history, and daily income.

**Conclusion:**

Work involving e-waste is associated with a higher prevalence of respiratory symptoms and with an increased risk of FEV1 and FVC decline, as well as of lung function impairment, particularly of restrictive disorders. Further studies to better clarify this association are needed. Awareness on this major public health threat should be raised in other sub-Saharan and Asian urban areas.

## 1. Introduction

In recent decades, scientific and technological advances have significantly improved lifestyles in populations globally. This unfortunately results in the growing production of an important amount of wastes, particularly from electrical and electronic equipment, also called e-waste [1].

Overall, e-waste refers to various chemical components such as lead, chromium, cadmium, mercury, and brominated flame retardants. These products are highly toxic for the whole environment and are responsible for human health effects [2, 3]. There are reports in the literature which have demonstrated associations between estimate exposures to these chemical products and disorders on the respiratory system, such as impaired lung functions, asthma, chronic obstructive pulmonary disease (COPD), and cancer [4, 5]. e-Waste may also affect several other organs or systems, such as the heart, genital organs, and the endocrine system [6].

Annually, several million tons of e-waste are thought to be generated worldwide [1, 7]. Western and Asian countries are at the top in both producing and consuming e-wastes in the world [1].

However, recycling of such chemical toxic products remains a major challenge and is hardly carried out, despite the growing global e-waste production [7]. Although Sub-Saharan Africa only accounts for 5.4% of the e-waste production worldwide, this part of the world has been playing a major role in the flow of the global e-waste [8]. Moreover, West African citizens are becoming second-class consumers for many rejected products [6], while some are recycled at lower cost and usually in precarious and deplorable hygiene, safety, and sanitary conditions [7, 8].

Irrespective of the 1992 Basel Convention clauses [9], the European continent continues to discharge the hazardous waste to Africa, to avoid or reduce costs required for their proper handling. For instance, one of the well-known e-waste sites in the world, Agbogloshie, also called “Europe's electronic dustbin”, is located in the Accra suburbs in Ghana [10]. Waste is dumped in the open air, and e-waste workers rush in, without any protection, in the goal of retrieving any potentially relevant materials for their professional activities [8].

The risk to health of e-waste-related exposures to organic and inorganic chemicals is rightly worrying among workers, who are directly involved in the recycling activities, especially when this is achieved without any protection [11], and this is worsened by the informal nature of these activities for many workers, who, additionally, are unaware of the dangers [12].

More importantly, e-waste recycling workers are not the unique affected by this issue, since surrounding populations are also potentially exposed to the diffusion and propagation of the generated pollutants and their consequences for the environment [7, 13].

In the West Africa, all countries are affected by the issue of e-waste handling at various levels, including Ghana and Nigeria [4, 14, 15]. However, very few studies on this major health public concern were carried out in the past in these settings and only focused on general health disorders, not respiratory damages.

Using data from Benin, the current study tended to estimate for the first time the frequency of respiratory symptoms and lung function disorders among workers involved in e-waste recycling in the informal sector in the city of Cotonou.

## 2. Methods

This was a cross-sectional study that was conducted in Cotonou, the largest city in Benin Republic, where most of the economic activities of the country are based. The study was conducted from August 14th to October 16th 2020.

Exposed participants were recruited among e-waste workers from a sampling frame obtained after a preliminary survey identified the number of e-waste sites in the city. A stratified sampling method by proportional probability was used, with respect of the different categories of e-waste workers: collectors, repairers, and dismantlers. Within each stratum, a list of e-waste workers was drawn up by the research team. Targeted participants were then randomly selected. Assuming a prevalence of respiratory disorders of 24.7% [16] in e-waste workers, an error *α* equal to 0.05 and a precision of 7%, the minimal sample size was estimated at 146.

Nonexposed participants were recruited from the general population among inhabitants who did not declare being involved in e-waste management. One nonexposed participant was matched with on exposed worker, based on age and sex.

Respiratory symptoms were investigated using an adapted British Medical Research Council's standardized respiratory questionnaire, which includes the following variables: wheezing, chest pain or tightness, shortness of breath, cough, and difficulty breathing or dyspnea [17]. Height and body weight were then measured in the upright position with as little clothing on as possible without shoes for all patients, followed by lung function testing (LFT) according to American Thoracic Society (ATS) recommendations, using the MIR SPIROBANK II portable spirometer [18]. LFTs were performed by a trained field epidemiology survey investigator. The spirometer was calibrated on a daily basis. A bronchodilator reversibility test was systematically performed using 400 micrograms of salbutamol with an spacer device according to the ATS/ERS guidelines [18]. Validation criterion of the test were as follows: (i) a “plateau” of at least one second at the end of the test, (ii) exhalation time of at least six seconds, and (iii) an absence of artifacts. A period of at least 30 seconds was observed between two maneuvers. A maximum of eight maneuvers was performed in each participant, and the three best maneuvers were deemed acceptable.

The largest FVC and FEV1 obtained from any acceptable curves were considered for this study. The criterion used to define LFD is summarized in Table 1[18].

Nutritional status was categorized in four groups: underweight (BMI < 18.5 kg/m^2^), normal (18.5 ≥ BMI < 25 kg/m^2^), overweight (25 ≥ BMI < 30 kg/m^2^), and overweight (BMI ≥ 30 kg/m^2^).

Data were collected directly with a tablet using the KoboToolBox application and analyzed using Stata V15 software (StataCorp. 2017. Stata Statistical Software: Release 15. College Station, TX: StataCorp LLC). Categorical variables were described by proportions and were compared between the two groups using the Chi^2^ (or Fischer exact test as appropriate). Continuous variables were described using means and standard deviation (SD) and were compared with *t*-test. In order to assess the effect of exposure to e-waste on LFT parameters (FEV1 and FVC), we performed a multiple linear regression adjusting for potential confounding factors. A value of *p* < 0.05 was considered significant.

### 2.1. Ethical Considerations

The study was conducted after formal approval by Ethics Committee of “Institut des Sciences Biologiques Appliquées” of Cotonou, Benin. Data were collected anonymously. Study databases were kept confidential and were protected with a password, only accessible to key persons involved in the study. Study participants gave written informed consent.

## 3. Results

### 3.1. Basic Characteristics

One hundred and forty-eight participants were included in each group, making a total of 296 participants. The mean age of participants was similar in both groups and was 29.7 ± 9.4 years in the unexposed group and 29.6 ± 9.8 years in the exposed group, with extremes ranging from 16 to 59 and 14 to 64 years, respectively. They were predominantly male (99.3% in each group). Participants with a daily income between $4 and $6 were the most represented (43.0%). Regarding anthropometric parameters, a normal body mass index (BMI) was found in 67.8% of the exposed subjects and in 75% of the unexposed subjects. Current smokers were significantly higher in the exposed compared to the nonexposed group, respectively, 15.5% and 2.0% (*p* < 0.001) (Table 2).

### 3.2. Respiratory Symptoms

The overall proportion of respiratory symptoms was higher in the exposed group compared to the unexposed group (33.1% vs. 21.6%, *p* = 0.027). The most reported symptom in both groups was cough (13.5% vs. 8.8%) but the difference was not significant (*p* = 0.196). Compared to the unexposed group, participants potentially exposed to e-waste due to their handling work during recycling that showed higher proportion of chest tightness (11.8% vs. 2.7%, *p* = 0.003) and breathlessness (6.8% vs. 1.4%, *p* = 0.018). Proportions of wheezing were not significantly different between the two groups (Table 3).

### 3.3. Lung Function Disorders

Comparison of the main spirometric parameters showed a significant difference between the mean FEV1 (±SD) of exposed (3.3 L ± 0.5 L) and unexposed participants (3.7 L ± 0.7 L), *p* < 0.001. Similarly, there was a significant difference between the mean FVC (±SD) of exposed (3.6 L ± 0.6 L) and the one of unexposed participants (4.3 L ± 0.8 L), *p* < 0.0001 (Table 3). In terms of lung function disorders (LFD), exposed participants showed a higher proportion than unexposed participants (25.0% vs. 14.9%, *p* = 0.029). There were also higher proportions of RVD and MVD in the exposed group of participants compared to unexposed matched controls, respectively (10.8% vs. 2.7%, *p* = 0.005) and (4.1% vs. 0.0%, *p* = 0.013). No difference was found in proportions of OVD between the two groups (Table 4).

After multiple linear regression and adjustment for age, BMI, daily income, asthma history, and smoking status, handling or working with e-waste was associated with a significant decrease of FEV1 by 0.42 L (95% CI: 0.29 L-0.55 L; *p* < 0.001) and of FVC by 0.75 L (95% CI: 0.59 L-0.91 L; *p* < 0.001) (see Table 5).

## 4. Discussion

Findings from this study provided for the first time in Cotonou, Benin, useful information on the symptoms of respiratory disorders among workers exposed to e-waste during handling or recycling electronic equipments. This finding is consistent with data from previous studies. For instance, in one study from Guiyu, South China, Zheng et al. reported a significantly higher prevalence of respiratory symptoms in exposed subjects compared to the unexposed group, respectively, 71.6% and 31.2% [5]. The higher prevalence of respiratory symptoms found in this study in China compared to our findings may be explained by regional variations in overall air quality and e-waste imports. Contrary to Cotonou, China, and other Asian settings are known as megalopolis for the deposit and informal e-waste recycling for many years, with as result, a more polluted and contaminated environment (including water, air, and soil). The differences may also be related to specific population characteristics and other environmental and domestic concomitant exposures.

When specifically focusing on the reported symptoms, coughing was found in more than 1-in-8 e-waste workers, chest tightness in about 1-in-11, and breathlessness in approximately 1-in-15. The prevalence of chest tightness and breathlessness was higher in the exposed group than among control participants. In comparison with data from the literature, these proportions sometimes vary, depending on the study sites and populations. For instance, in one study from Ghana, a nearby West African country, the proportion of the different respiratory symptoms among e-waste workers was much higher than that found in the current study and reached 37% for cough and 45.5% for chest tightness [19]. The inclusion of burners in the Ghana study may be one possible reason, through continuous smoke inhalation and chronic inflammation of the bronchial mucosa, while in Cotonou, Benin, participants potentially exposed were predominantly collectors, repairers, and dismantlers. Unsurprisingly, as reported from elsewhere, coughing has been the most expressed symptom reported, since this represents the most common manifestation in case of any respiratory concern [19, 20]. The higher prevalence of chest tightness and breathlessness in the exposed group compared with the unexposed participants was also observed in a precedent study from China [13].

Exposure to e-waste is not exclusively limited to workers in the informal sector. Those who are formally employed are also potentially exposed and do not spare its harmful damages. In one systematic review, exposure to metals in a formal sector has been demonstrated to exceed the recommended threshold [21]. Likewise, brominated flame dosage in inhaled air as well as biological samples of workers has shown higher levels compared with reference groups [21]. Local residents dwelling around the e-waste recycling zone, including children, are also at increased risk of carcinogenic metals inhalation, cancers, and other diseases [13].

With respiratory symptoms, LFD are the other abnormalities that were investigated during this survey. Other studies have suggested that e-waste may induce LFD [2, 3, 13]. Our findings suggest a significantly higher proportion of probable RVD among e-waste workers. This is consistent with that reported by Huang et al. who also showed a significantly higher proportion of RVD among e-waste workers [22]. The difference found with MRD seems to stem from that observed with RVD.

Similar to these disorders, analysis of relevant LFT parameters concluded to a significant reduction of FEV1 and FVC in exposed compared with unexposed participants, and this remained consistent despite adjustment with other potential confounders such as age, body mass index, and smoking.

Nti et al. in a longitudinal study showed a consistent decline of FEV1 among all e-waste workers, especially in burners, after adjusting for seasonal variation, age, and cigarette smoking [19]. This is probably due to the well documented relationship between PM2.5, PM2.5-10, PM10, and lung function decline or chronic respiratory diseases [20]. Other authors even demonstrated a specific link between PM2.5 exposure and RVD [22]. PM production from e-waste leads to its deposition along the respiratory tract and its accumulation in the pulmonary alveoli and thickening of the alveolar-capillary wall [2, 3]. This probably explains the predominance of RVD among e-waste workers [13].

Additionally, pollutants other than PM produced during e-waste recycling activities, mixed with other inhalation exposures such as gaseous, polycyclic aromatic hydrocarbons, and heavy metals, have been demonstrated to be associated with the development of lung function impairment [19].

Our study has some limitations. First, the exposed group may have some background exposure to e-waste in their living environment; this could have led to an underestimation of the difference in respiratory symptoms and LFD between both groups. Second, we were not able to collect data neither on PMx exposure nor on the dosage of organic or inorganic pollutants in various blood, urine, and skin samples. Third, our study was only conducted in Cotonou, Benin, and may not be generalized to the whole country. Fourth, due to resources constraints and unavailability of diagnostic tools such as plethysmography, confirmation of RVD using total pulmonary capacity was not possible. Despite these limitations, this study raises awareness on the risk of workers handling e-waste in developing countries. Further investigations are required for better documentation of this public health threat, through longitudinal studies to refine the association between this exposure and respiratory disorders.

## 5. Conclusion

Work with e-waste and subsequent exposure to chemicals including metals in the informal sector leads to a significantly higher frequency of respiratory complaints, mainly chest tightness and breathlessness, and to a significant reduction of lung function parameters such as FEV1 and FVC, after adjustment for other respiratory confounders. Of lung function disorders, RVD was found to be the most common among e-waste workers, compared to an unexposed group. Additional studies would help to clarify all the determinants of the association between e-waste exposure and respiratory disorders. Finally, there is an urgent need to call for actions about this increasing public health concern in sub-Saharan Africa and Asian nation urban areas, with the involvement of academic and scientific institutions, the government, and other stakeholders.

## Figures and Tables

**Table 1:** Definition criterion of lung function disorders adapted from ATS/ERS consensus.

Abnormalities	Definitions
Obstructive ventilatory disorder (OVD)	FEV1/FVC < LIN with FVC ≥ LIN
Suggestive restrictive ventilatory disorder (RVD)	FEV1/FVC > LIN with FVC < LIN
Suggestive mixed ventilatory disorder (MVD)	FEV1/FVC < LIN with FVC < LIN
Significant reversibility	FEV1 and/or FVC increase by at least 200 ml and at least 12% from baseline after bronchodilation
Normal spirometry	FEV1/FVC > LIN and FVC ≥ LIN

FEV1: forced expiratory volume in one second; FCV: forced vital capacity; LIN: lower limit of the normal.

**Table 2:** Comparative baseline characteristic of e-waste workers in informal recycling sector (exposed group, *n* = 148) and matched unexposed subjects (*n* = 148) in Cotonou, Benin (*N* = 296).

	Unexposed, *n* (%)	Exposed, *n* (%)	All, *n* (%)	*p* value
Daily income ($)				0.007
<4	29 (22.7)	19 (13.4)	48 (17.8)	
4-6	47 (36.7)	69 (48.6)	116 (43.0)	
6-10	17 (13.3)	31 (21.8)	48 (17.8)	
>10	35 (27.3)	23 (16.2)	58 (21.5)	
Nutritional status^∗^				0.478
Undernutrition	5 (3.4)	6 (4.1)	11 (3.7)	
Normal	99 (67.8)	111 (75.0)	210 (71.4)	
Overweight	30 (20.6)	23 (15.5)	53 (18.0)	
Obesity	12 (8.2)	8 (5.4)	20 (6.8)	
Current smoking^∗∗^	3 (2.0)	23 (15.5)	26 (8.8)	<0.001
History of asthma	9 (6.1)	4 (2.7)	13 (4.40)	0.156
History of tuberculosis	1 (0.7)	0 (0)	1 (0.3)	0.316

^∗^Nutritional status was assessed with body mass index (BMI) in kg/m^2^ calculated as weight/(height)^2^; BMI < 18.5, undernutrition; ≤18.5 and<25, normal; ≥25 and<30, overweight; ≥30, obesity. ^∗∗^Smoking history in the past 30 days.

**Table 3:** Respiratory symptoms reported by e-waste workers in the informal sector (exposed, *n* = 148) compared to matched unexposed subjects (*n* = 148) in Cotonou, Benin (*N* = 296).

	Unexposed, *n* (%)	Exposed, *n* (%)	*p* value
Wheezing	6 (4.1)	9 (6.1)	0.427
Chest tightness	4 (2.7)	17 (11.8)	0.003
Cough	13 (8.8)	20 (13.5)	0.196
Breathlessness	2 (1.4)	10 (6.8)	0.018
At least one symptom	32 (21.6)	49 (33.1)	0.027

**Table 4:** Lung function disorders among e-waste workers in the recycling sector (exposed, *n* = 148) compared to matched unexposed subjects (*n* = 148) in Cotonou, Benin (*N* = 296).

	Unexposed, *n* (%)	Exposed, *n* (%)	*p* value
Obstructive disorders	18 (12.2)	15 (10.1)	0.248
Suggestive restrictive disorders	4 (2.7)	16 (10.8)	0.005^∗^
Suggestive mixed disorders	0 (0)	6 (4.1)	0.013^∗^
All disorders	22 (14.9)	37 (25.0)	0.029
Total evaluated	148	148	

^∗^Fisher exact test.

**Table 5:** Effect of estimated exposure to e-waste via handling work on FEV1 and FVC determined by multiple linear regression after adjustment on age, BMI, asthma history, and daily income in e-waste workers (exposed, *n* = 148) compared to matched unexposed subjects (*n* = 148) in Cotonou, Benin (*N* = 296).

	FEV1 (L)		FVC (L)	
	*β* (95% CI)	*p*	*β* (95% CI)	*p*
Exposed to e-waste	-0.42 (-0.55; -0.29)	<0.001	-0.75 (-0.91; -0.60)	<0.001
Age	-0.03 (-0.04; -0.02)	<0.001	-0.03 (-0.04; -0.02)	<0.001
BMI	0.009 (-0.09; 0.03)	0.324	0.02 (0.006; 0.04)	0.044
Smoking habits	0.04 (-0.18; 0.25)	0.741	0.14 (-0.12; 0.41)	0.301
Asthma history	-0.02 (-0.34; 0.30)	0.901	0.06 (-0.32; 0.46)	0.728
Daily income ($)				
<4	1		1	
4-6	0.09 (-0.09; 0.29)	0.316	0.13 (-0.1; 0.4)	0.262
6-10	0.16 (-0.06; 0.39)	0.152	0.28 (0.06; 0.56)	0.045
>10	0.26 (0.03; 0.50)	0.024	0.34 (0.07; 0.62)	0.016

BMI: body mass index.

## Data Availability

Data available upon reasonable request.
